# Defining mental disorder. Exploring the 'natural function' approach

**DOI:** 10.1186/1747-5341-6-1

**Published:** 2011-01-21

**Authors:** Somogy Varga

**Affiliations:** 1 Institute of Cognitive Science University of Osnabrueck Albrechtstr. 28. D-49069 Germany; 2 Center for Subjectivity Research University of Copenhagen Njalsgade 140-142 DK-2300 Copenhagen S Denmark

## Abstract

Due to several socio-political factors, to many psychiatrists only a strictly objective definition of mental disorder, free of value components, seems really acceptable. In this paper, I will explore a variant of such an objectivist approach to defining metal disorder, *natural function objectivism*. Proponents of this approach make recourse to the notion of *natural function *in order to reach a value-free definition of mental disorder. The exploration of Christopher Boorse's 'biostatistical' account of natural function (1) will be followed an investigation of the 'hybrid naturalism' approach to natural functions by Jerome Wakefield (2). In the third part, I will explore two proposals that call into question the whole attempt to define mental disorder (3). I will conclude that while 'natural function objectivism' accounts fail to provide the backdrop for a reliable definition of mental disorder, there is no compelling reason to conclude that a definition cannot be achieved.

## Introduction

As an important element in the ongoing efforts to develop the DSM-V, researchers have again scrutinised the concept of mental disorder and accentuate that his is both a crucial field and one that needs additional work [1,2]. This is no surprise. The concept of mental disorder is at the foundation of psychiatry. While adequately defining mental disorder is a notoriously thorny issue with far reaching implications for psychiatric research, diagnosis, socio-political interventions, at least since the publication of the DSM-III there has been a consensus of the fact that a convincing definition of mental disorder is required. In addition, for many, the credibility of psychiatry as a medical discipline to a certain extent depends on a convincing definition. On the background of the socio-political developments that the American Psychiatric Association (A.P.A.) faced during the 1970s the effort in the DSM-III and DSM-IV to define mental disorder becomes more transparent [3,4]. First, proponents of the "anti-psychiatry" were radically questioning the scientific legitimacy of psychiatry: Sceptics like Szasz [5] were arguing that psychiatry, rather than being a genuine branch of medicine, is a masked form in which social power is exercised. Simply, the exercise of power works through a medicalisation of 'problems in living' that - following Szasz - are really non-medical in nature. Second, the classification of homosexuality as a mental disorder, as proposed by the DSM-II, became increasingly untenable. Third, support groups have worked for the recognition of mental disorders as a disease like others (diseases of the brain) to lessen stigmatisation and to secure the same benefits to the mentally ill as to the physically ill.

Against the background of these issues that psychiatry was struggling with, it is comprehensible that only a value-free definition of mental disorder seemed really acceptable. The idea is that if mental disorder could be defined relying solely on 'pure' facts free of value judgments then psychiatry would no longer be vulnerable to anti-psychiatric attacks. There are of course varieties of such 'naturalism' about defining mental disorder. First, in the framework for studying mental disorder known variously as the 'biomedical', or 'disease' model, mental disorder is often understood as a 'natural kind' Zachar [6,7]. I have elsewhere dealt with the pros and cons of such a view (X forthcoming), and in this paper, I will consider a second version of such naturalism, which I suggest we term *natural function objectivism*. Proponents of this view hold that while evaluation does trigger research interest, but that after this preliminary evaluation the further study into the condition can be done objectively, without the involvement value-laden judgments. Value-dependent evaluations are not important to settle the issue whether something is dysfunctional. Rather, in order to objectively identify a particular condition as a disorder, proponents of this approach make recourse to the notion of *natural function*. As I see it, *natural function objectivism *comes in two varieties depending on the way the 'naturalness' of natural functions is understood. Guided by this distinction, the paper falls into two parts, dealing with the work of two authors that are generally agreed on as the most prominent proponents of each view [1]. In the first part of the paper, I will explore Christopher Boorse's 'biostatistical' account of natural function that builds on a combination of biological function and statistical normality (1). In the second part, I will investigate the merits of the '*hybrid naturalism' *approach to natural functions, originally developed by Jerome Wakefield. This account is hybrid because it both accepts a value component (harm), while still embracing an objective, evolutionary account of natural functions. The objectivist dimension in this account is that psychological sub-systems that constitute a human being were naturally selected to perform certain functions and that such natural functions are objectively discoverable (2). The goal of the first part will be to critically assess the arguments supporting the idea that we should conceptualise psychiatric disorders in recourse to such accounts of natural functions. To anticipate a central point, I will argue that due to decisive difficulties of such natural function accounts they fail to provide the backdrop for a reliable definition of mental disorder. Then, in the third and last part of the paper I will argue that the seemingly unsolvable classificatory problems have led to two proposals that to an extent might be understood as echoing a certain resignation. One argues that we should give up the idea of a strict definition of mental disorder, because it is impossible to provide necessary and sufficient conditions for membership in the category 'mental disorder.' Another proposes that we should re-map definitions depending on psychopharmacological effects. I will counter these strategies and argue for the possibility of a definition (3).

As mentioned, within the framework of the development the DSM-V, researchers have once more directed focus on the concept of mental disorder. A crucial issue in this discussion is whether the concept of dysfunction, which emerges out of the naturalistic approaches that I deal with in this paper, should be retained in the DSM-V. There is considerable disagreement on this issue [1,8] and a clarification is needed. While not providing a definition, I expect that the findings of this paper will contribute to the advancement the debate towards a more valid and philosophically robust definition.

### 1. Biostatistics: Biological Function and Statistical Normality

Scadding [9] has influentially defined disease as "the sum of the abnormal phenomena displayed by a group of living organisms in association with a specified common characteristic or set of characteristics by which they differ from the norm for their species in such a way as to place them at a biological disadvantage." Recently, in their widely used book *Models for Mental Disorder. Conceptual Models in Psychiatry*, Tyrer & Steinberg [[10], p. 8) embrace this definition, holding that it is equally suited to help define both physical and mental disorder. The authors remark such a statistics-based definition of mental disorder would harbour decisive advantages: it is held that such a model does not have to involve value judgments in order to define mental disorder, and therefore one of the gravest problems of a definition could be solved. Additionally, it is argued that such a definition might set limits to what critics have called the growing medicalisation of 'problems in living'. This is, because in order for something to qualify as a disorder, it has to be both statistically deviant and biologically disadvantageous. This limiting aspect ensures that conditions such as 'late luteal dysphoric disorder' (premenstrual tension) would not count as genuine disorders. While such a condition might deviate from the norm, in this view it cannot qualify as a disease, because it cannot be shown to be directly disadvantageous. So due to the fact that it is only a necessary but not a sufficient condition for a disorder to differ from the statistical norm, Tyrer & Steinberg are confident that such a definition is immune to accusations that psychiatry treats 'problems in living'. As they note "The disease model in psychiatry helps to decide which of these conditions is beyond its scope and unsuitable for mental health interventions" [[10], p. 8]. Along the same lines of thought, Kendell [11] has embraced a comparable biostatistics-informed notion of disease, arguing that manic depression and schizophrenia can legitimately be classified as diseases because sufferers can be shown to have a biological disadvantage: they live shorter lives and have fewer children than the rest of the population. However, as Cooper [4] points out, the claim that disorders are conditions that reduce life expectancy or fertility are untenable. These are neither necessary nor sufficient conditions for a disease. Being a professional mercenary is statistically infrequent and most likely reduces life expectancies, but this does not warrant the label disorder. It is equally implausible to claim a necessary link between health and fertility.

Christopher Boorse has proposed a more complex and influential biostatistical account of disease that provides a solution to some of these problems. Although often Boorse discusses physical disorders, he also explicitly takes his account to apply to mental disorders [12]. Boorse holds that the definition of metal disorder and psychiatric classification in general can indeed be scientific and objective, because it is possible to detect a disorder solely by bio-statistical means and without any recourse to value judgements. He argues that the value-neutrality of a classification can be maintained if a definition of mental disorder is informed by our knowledge of biological design: "If diseases are deviations from the species biological design, their recognition is a matter of natural science, not evaluative decision" [[13], p. 543]. He has recently re-confirmed his position, maintaining that "disease is only statistically species-subnormal biological part-function; therefore, the classification of human states as healthy or diseased in an objective matter, to be read off the biological facts of nature without need for value judgments" [[12], p. 4]. Just as in Tyrer & Steinberg [10] and Scadding [9], Boorse's biostatistical theory "rests on the concepts of biological function and statistical normality" [[12], p. 4]. Thus, just as in Tyrer & Steinberg and Scadding, Boorse defends a naturalist position and maintains that value-judgments can be omitted if one focuses on the 'natural fact' that underlies a mental disorder.

Boorse's Bio Statistical Theory (BST), equates health with normal species functioning, which is the *statistically typical *contribution of a sub-system to the organism's overall goals: survival and reproduction. The group with respect to which a contribution is statistically typical is the *reference class*, a group made up of all the individuals belonging to the same age group, sex and race of an individual [13]. Compared to the accounts of Tyrer & Steinberg and Scadding, the introduction of a reference class is one of the genuine achievements of Boorse. At the same time the idea of reference class provides a solution to the problems that Tyrer & Steinberg and Scadding ran into. So a person is healthy if all the sub-systems that constitute him function in ways that are statistically typical for the reference class of the person that entails race, age and gender. Boorse maintains that reference classes are needed due to the vast variety of functioning. In many cases, it is impossible to identify variables that are statistically typical for the *entire *species. For example, the same hormonal levels could mean both health and disease depending on the age and gender of the person. But on the other hand, the reference classes cannot go below a certain size: if the reference group for healthy liver functioning is made up of men with a high alcohol consumption, then liver functioning indicators that we normally would consider as diseased, would appear as within a statistically normal range. In other words, it seems that the BST hinges on an account of the kind of reference classes: disease is not to deviate from any, but the 'appropriate' reference classes only. Therefore, Boorse must justify his choice of reference classes and show that the distinction between appropriate and inappropriate reference classes rest on neutral, empirical facts. In the work of Boorse, this question of justification is solved in the following manner: the appropriate reference class is simply conceptualised as "a natural class of organisms of uniform functional design" [[13], p. 562]. In other words, for Boorse, the reference class rests on neutral, natural facts. The term 'functional design' might evoke the thought that Boorse is attempting to justify his reference classes by recourse to evolutionary biology. However, Boorse explicitly rejects such a strategy of justification [14,15]. While Boorse's account certainly has some merits compared to the ones proposed by Tyrer & Steinberg and Scadding, in the following I will argue that Boorse's solution harbours three problems, pertaining to issues of reference class (a), circularity (b) and value (c).

#### a) Reference Class

As I have noted, since the label 'disorder' is only warranted by the deviation from 'appropriate' reference classes, the account hinges on whether Boorse is able to give appropriate justification for his conceptualisation of the 'appropriate' reference classes. It is not difficult to see that it is lastly the reference class that determines the boundary between disease and health. And here, some problems arise: if a definition of disorder is not to make recourse to evaluative judgment, Boorse has to show that the distinction between appropriate and inappropriate reference classes rest on neutral, empirical facts. In the work of Boorse, this question of justification is solved in the following manner: the appropriate reference class is simply conceptualised as "a natural class of organisms of uniform functional design" [[13], p. 562]. In other words, for Boorse, the reference class rests on neutral, natural facts. But obviously, 'natural' here cannot be taken to refer to whatever occurs in nature, because obviously both appropriate and inappropriate ones occur in nature. Rather than just 'occurring in nature', the criteria of defining appropriate reference classes are mixed: some characteristics that define appropriate reference classes are innate (like sex and race), while others (like age) are clearly acquired. So the question arises: if 'natural' is not taken to refer to whatever occurs in nature, what then justifies the 'appropriateness' of Boorse's reference classes? As I see it, although Boorse's account hinges on a justification of appropriate reference classes, he nevertheless fails to provide such justification. Due to this lack, the problem that remains is quite striking. For instance, if I describe myself as Caucasian, blue-eyed and tall, in Boorse's account no empirical facts that determine that only 'Caucasian' would count as the appropriate reference class. In summary, Boorse fails to provide an objective justification for his selection of reference classes and thereby an objective justification for his concept of disorder.

#### b) Circularity

In an important manner the BST is vulnerable to charges of circularity. The goal for Boorse is to distinguish healthy from diseased functioning by appealing to a standard of normality. We should keep in mind that Boorse aims to explain normality in terms of the design of the organism: The normal is "to be defined as that which functions in accordance with its design." In his non-evolutionary framework the criteria for determining what something's design or function is that it has to make "a standard causal contribution to a goal actually pursued by the organism." These goals can be identified empirically, (statistically) "without considering the value of pursuing them". In other words, studying the statistically normal behaviour of the organism can identify the goals of the organism. Now it seems that Boorse has moved in a circle, since statistic normality is being employed to define functioning according to design, which then is used to elucidate the notion of normal [16]. So it seems that the two central terms for Boorse are explained by reference to each other. Somewhat simplified the two claims are this:

1) Whether X is a function is determined by whether X is normal behaviour

2) Whether X is a normal behaviour is determined by whether X is a function

In the first case, Boorse claims that in order to identify X as a function, it needs to be established that X is a normal behaviour. At the same time, according to the second claim, normal behaviour is determined by function. So the problem of circularity arises when function (qua causal contribution to a goal) is determined by normal behaviour, and at the same time normal behaviour is determined by function. Instead of referring to each other and to live up to Boorse's claims, the two concepts must be explained independently.

#### c) Value-ladeness

According to Boorse, one of the main advantages of the BST would be that it could provide a value-free definition of disease. But let us again raise the issue of homosexuality. Then, the BST should be able to tell us whether such a condition qualifies as a disorder. As Cooper [[4], p.17] rightly notes, someone who endorses Boorse's account is forced to accept that homosexuality might be a disorder. Boorse has more recently accepted this result, but defends his account by saying that a determination of a condition as a disease does not imply that it should be treated by health professionals [[12], pp. 11-12]. But in this case, the price is that the account of disease is deflated, and quite far from our normal use of the concept of disease, which includes that a disease is actually a bad thing to have. On the other hand, as previously mentioned, the question whether homosexuality is a disease can be reduced to the question whether sexual orientation should count as an appropriate reference class. If sexual orientation counts as an appropriate reference class, then homosexuality is not a disease, whereas if sexual orientation does not qualify as an appropriate reference class, homosexuality is a disease. But as we have seen, the issue of appropriate reference classes cannot be solved by drawing on empirical facts: the judgment about what should count as an appropriate reference class precedes the empirical realm. And since no empirical fact can be deployed to identify whether sexual orientation should not qualify as an appropriate reference class, no empirical fact can determines whether homosexuality is a disease.

To conclude, Boorse's connection of abnormal function to statistical deviance is problematic. The crucial issue of how to define natural function is not adequately solved. How is natural, species-typical functioning to be grasped that is adequate to the organism's age and contributes to survival and reproduction? Maybe therefore, in order to build a stronger account of function, in his reply to criticism he attempts to include evolutionary explanations of function in account, which is clearly inconsistent with his rejection of evolutionary terms [[15], p. 135]. Now, statistically normal functioning in a species is understood as a marker of the evolutionary design. With this move Boorse shifts the terrain, since now the question is how to define function in evolutionary terms, thus how to determine whether a conditions assists or obstructs the continuance of the species. While I shall address this approach in the following, let me already mention that such a solution is deemed to fail: evolutionary dysfunction is neither necessary nor sufficient for something to be a disease.

An influential account that deploys a notion of natural function informed by evolutionary theory has been proposed by Jerome Wakefield. In the next section I will deal with such an evolutionary-based account of function and while exploring this position, I will attempt to exemplify the consequences of evolutionary approaches function to depression.

### 2. "Hybrid" Naturalism

In a series of influential papers, Wakefield has persuasively maintained that a condition should count as a disorder if and only if it is a harmful dysfunction. In other words harm to the individual is a *necessary condition for *a disorder: a condition cannot be a mental disorder unless this condition "causes some harm or deprivation of benefit to the person as judged by the standards of the person's culture" [[17], p. 385]. In contrast to Boorse, besides incorporating a factual component (dysfunction), Wakefield's account also adds an important value component (harm). With this move Wakefield creates a "hybrid" definition that tries to bridge the gap between the previously incompatible positions: the "social-constructivist" position that understood mental disorder as a value-laden social construct and the position that defined mental disorders as natural entities to be understood in biological terms [18].

"I have proposed a hybrid account, the "harmful dysfunction" (HD) analysis of the concept of mental disorder. According to the HD analysis, a disorder is a harmful dysfunction, where "harmful" is a value term, referring to conditions judged negative by sociocultural standards, and "dysfunction" is a scientific factual term." [[19], p. 149]

"A condition is a disorder if and only if (a) the condition causes some harm or deprivation of benefit to the person as judged by the standards of the person's culture (the value criterion), and (b) the condition results from the inability of some internal mechanism to perform its natural function, wherein a natural function is an effect that is part of the evolutionary explanation of the existence and structure of the mechanism (the explanatory criterion)" [[17], p. 384].

So the claim of a factual malfunctioning of an internal mechanism that causes objective dysfunction is paired with the claim the social definition of "harm" depends on the cultural context. What I would like to emphasise in this context is within the hybrid construction, Wakefield nevertheless remains objectivist about natural function. The underling claim is twofold: first, psychological sub-systems that constitute a human being were naturally selected to perform certain functions, Second, such natural functions are objectively discoverable. In this manner, he embraces an evolutionary account to define dysfunctions as "failures of internal mechanisms to perform the functions for which they were naturally selected." Recently [[19], p.149] Wakefield has restated his point adding that "in modern science, "dysfunction" is ultimately anchored in evolutionary biology and refers to failure of an internal mechanism to perform one of its naturally selected functions." So while he accepts that for a condition to be a disorder it is necessary that the dysfunction is harmful, he claims that there has to be an evolutionary dysfunction in order to qualify as a disorder. Incorporating both value and scientific criteria, Wakefield's hybrid account is an attractive alternative, but nevertheless, in the following I will point out three problems with his version of naturalism about natural function, pertaining to statistical norm (a), evolutionary mismatch (b) and adaptively neutral exaptations (c).

#### a) Statistical norm

In a sense, Wakefield runs into the problem that haunted Boorse's account. Like Boorse, Wakefield accepts a dimensional view of disorder, in which dysfunction is not a categorical break, but a graded deviation. The backdrop against which such a deviation can be identified is in Wakefield's case an evolutionarily designed natural function. But then, the same question that haunted Boorse's account also inescapably pops up here: how can a non value-laden dimensional boundary between function and dysfunction be created? In principle just like Boorse, Wakefield must also identify the evolutionarily designed response to the environment via recourse to a statistical norm: thus, the designed response is identical to what is exhibited by the majority of the population. There are two problems with this move. First, as Lilienfeld & Marino [20] have noted, such an approach does not take into account that natural selection often produces substantial variability across individuals. Second, when attempting to distinguish genuine disorders from normal responses, at least sometimes Wakefield makes recourse not to evolutionary psychological theory, but to an estimation about appropriate response. Let me try to demonstrate what this amounts to. In the case of depression, among the DSM-IV criteria for major depressive disorder there is an exclusion for uncomplicated bereavement: following loss of a loved one, up to two months of depressive symptoms are regarded as normal. In Wakefield's account [21], such a reaction might be a designed response since it represents a reaction that is exhibited by the majority of the population. This is not to suggest that Wakefield subscribes to the DSM IV criteria for Major Depressive Disorder. In fact, Horwitz and Wakefield [18] argue at length that the DSM IV criteria are too inclusive, resulting in the 'Loss of Sadness'. But all things considered, the evolutionarily designed response to the environment is found via recourse to a statistical norm. To be clear, I am not denying that a certain depression-like reaction might be a designed response. Also, it might be the case that such designed responses can be identified by statistical means. However, what I wish to emphasise here, is that there is no objective standard to determine whether "up to two months" represents an evolved response to environmental stress. What adds to the implausibility of such a way of reasoning is the well-known fact that adaptive responses differ across individuals.

#### b) Mismatch explanations

One of Wakefield's important assertions, namely that important mental and physical systems were most likely designed by evolution to perform a given function, is highly problematic. At least a number of systems cannot be adequately conceived of as selected, fitness-increased adaptations. Rather, they might be 'exaptations' that fall in two kinds. First, there are 'exaptations' that are secondary adaptations: the feather system of birds is a good example here. It is widely held that the feather system originally evolved to enable heat regulation in birds, while it was only in a secondary process that it became an instrument for flight. So such secondary exaptations have enhanced fitness at the point of their emergence, but might have since taken on fitness-increasing functions that are dissimilar from their original functions. Given the definition of *dysfunction *as the failure to fulfil originally designed function, Wakefield's account cannot explain secondary adaptations. A broken wing of a bird might still fulfil its original function as a heat regulator. Surely, if one modified the account so that it only refers to current design, then it could accommodate secondary adaptations. But, as Lilienfeld & Marino [20] have shown this would lead to other unsolvable problems.

Let us see what the consequences of Wakefield's account would amount to in the case of depression. For many, since depression is suitably prevalent through history it is seen to be appropriate to investigations of its evolutionary origins. But first, it is useful to point out a helpful taxonomy of evolutionary explanations by Murphy [22] that distinguishes between three types of evolutionary explanation of psychopathology. A *breakdown explanation *understands mental disease as the malfunction of some component of the mind/brain in fulfilling its evolutionary function. A *mismatch explanation *understands mental disease as connected to a mechanism that was once adaptive but is no longer adaptive because of changes in the environment. So the pathology lies not in some sub-system of an individual, but in the mismatch between the ancestral environment and our current environment. A more controversial *persistence explanation *holds that some putative disorders qualify as adaptive even in the present environment. So far, such evolutionary-informed investigations into depression have tended to follow two opposing paths: *mismatch explanation *and *persistence explanations*. Both build on the idea that those mechanisms or devices activated in depression evolved to manage hostile situations in which flight was impossible. From here, the two accounts part ways: The so-called "dysregulation" model, defended by Nesse [23] and Gilbert and Allan [24], hypothesises that in depression this inherited mechanism becomes pathologically over-activated. One seriously inhibiting aspect about the dysregulation model is that it does not address the individual and social factors, and thus does not address why some rather than others are affected.

What I wish to point out here is that the *mismatch explanation *of depression, if true, would challenge Wakefield's position. Price et al. [25] and Nesse and Williams [26] argue that depression should be understood as an evolved, adaptive response to specific problems that arise in the small, status-oriented social groups of our ancestors. So if depression can be understood as adaptive, it is not to the natural, but to the social environment. Gilbert [27] notes that it is not a loss of control over the environment that is particularly depressing, but the loss of control in the social environment. More specifically, depression is an adaptive response to the loss of status in such small social groups, that helps to accept lowered rank once having lost status [25,28]. Once out-competed, it is of advantage to self-evaluate, ruminate upon weaknesses and alter previous behaviours. The idea is that depressed mood helps to accept status loss and motivates the person to alter previous behaviours in order to ameliorate reproductive chances. The suggestion is that while in such small groups this adaptive response might have been a fruitful strategy resulting in social success, in contemporary and much more sizeable groups, this strategy is a mismatch. Due to a radically changed social setting the inherited mechanism - activated when one thinks that he is out-competed - is no longer adaptive, since it will be activated frequently in the modern world, as our peer group with whom we compete is tremendously larger. Such a mechanism will not only fail to accomplish the goal it was selected to achieve: given modern societal framework, the depressed affect-lowering response to change is not only ineffective but also seriously inhibiting and fitness decreasing. Nesse [29] takes this mismatch to explain at least to a certain extent the alleged worldwide rise of depression.

The mismatch explanation of depression challenges Wakefield's account, because it shows that the depressive responses can be seen as the results of systems performing their originally evolved functions, but in a mismatched context. If one is to incorporate such mismatched responses, then the problem that Boorse's account could not solve re-emerges: we would need to specify what level of depressive reaction should count as appropriate or inappropriate to be regarded as dysfunctional. As previously noted, there are no unambiguous ways of doing this, since there is no non-situational and value-free boundary separating dysfunction from normal functioning.

#### c) Adaptively neutral exaptations

Additionally, there are also *adaptively neutral exaptations *[20], non-selected features that are merely by-products of adaptations that have not enhanced fitness at the point of their emergence. Gould [30] has argued that the large size if the brain, the most complex and flexible organ, originally arose as an adaptation for some functions in humans' ancestral past, and the sheer complexity of it produced many by-products. Thus the human brain "throws up spandrels by the thousands for each conceivable adaptation in its initial evolutionary restructuring" [[30] p.58]. Good examples of such adaptively neutral exaptations that are unlikely to be of direct relevance to increase fitness are on Gould's account psychological capacities like religion, reading, writing, fine arts, the norms of commerce, arithmetic ability, music, or motor skills. More precisely, it has since been argued that these exaptations might be indirect, domain-general consequences of natural selection, like general intelligence [31,32]. Accommodating such exaptations is a problematic issue for Wakefield's account. For example people with congenital amusia, which is an established learning disability for music, a deficit that appears highly specific to the musical domain [33]. Afflicted people are unable to discriminate the pitch of two successive tones, to recognise familiar melodies or to remember a tune. Now, on Wakefield's account, since the impaired function is not the outcome of evolution, amusia would not count as a disorder.

All in all, it seems that dysfunction in evolutionary terms is simply neither necessary nor sufficient for disorder. Wakefield's account cannot accommodate *adaptively neutral exaptations *and *mismatches *(mechanisms once adaptive, but no longer adequate due to environmental changes). The upshot of this discussion is that in general, it is not the case that a disease must involve evolutionary dysfunction. If one is to hold on to an evolution-informed account of dysfunction and mental disease, one must identify the characteristic selection pressures of the time period when a certain function is taken to be adaptive. As we have seen, the same condition might be a function (in a time with certain social constellations responding to selection pressures) and at a later point a mismatched dysfunction.

So to conclude, this third part of the paper was devoted to the *hybrid naturalism *approach to defining mental disorder, that both accept a value component (harm) and embrace an objective, evolutionary-informed account of natural functions. Central to such a hybrid objectivist approach is the idea that the *natural function *of psychological sub-systems is determined by natural selection and that such natural functions are objectively discoverable. One might argue that Wakefield only says that whether something is a natural function is a factual matter. As he argues in "Metal Disorder as a Back Box Essentialist Concept", natural functions might be hidden in the 'black box'. However, even though the 'black box' idea might give rise to such an interpretation, Wakefield is in the same paper actually quite unequivocal that natural functions are objectively discoverable. As he notes: "Evolutionary theory (...) explains biological design and thus shows that *disorder *refers to a scientifically identifiable phenomenon" [[34] p. 465]. An important conclusion of this part was that dysfunction in evolutionary terms is simply neither necessary nor sufficient for something to be a disorder. Due to the severity of these problems, we have good reasons to reject this particular approach to defining mental disorder. Although the idea of a purely objective definition of mental disease appears attractive to many psychiatrists, the approaches explored in this paper fail to bring about such a definition.

### 3. Other strategies: Roschian concepts and psychopharmacological taxonomy

Perhaps also as a reaction to the debate about classification and to the severe problems with such 'objectivist' definitions of mental disorder, two proposals have emerged that radically call into question the whole effort of defining of metal disorder. In a sense, these proposals to a certain extent echo a resignation. The first account I wish to explore at this point draws on the idea of 'Roschian concepts' to argue that it is in principle impossible to provide necessary and sufficient conditions for membership in the category 'mental disorder' (a). The second account that I will address suggests that instead of conceptual philosophical and conceptual issues, a definition of mental disorders should be based on psychopharmacology. Roughly, the underlying idea is that a mental disorder is a condition that psychopharmacological products have alleviating effects on (b). In the following, I will argue that both of these strategies have crucial flaws and that they do not provide convincing reasons to reject the possibility of a good definition of mental disorder.

#### a) Roschian concepts

Scott Lilienfeld and Lori Marino [20] have suggested that the failure of a good definition is not a practical, but a principal problem. The authors claim that 'mental disorder' is a 'Roschian concept', characterised by the fact that it is in principle impossible to provide necessary and sufficient conditions for category membership. In a same way Mackinejad and Sharifi [35] have proposed a view of mental disorder as a Wittgensteinian family resemblance concept. Wittgenstein maintained that no necessary and sufficient conditions can be given for something which is a game. Rather, akin to members of a family, games are held together by a set of similarities, in a way that while all members of the family will not share the same feature, any two members will be similar in at least one way. Instead, Scott Lilienfeld and Lori Marino [20] propose that for a condition to qualify as a mental disorder hinges on grades of similarity to prototypical cases. While one cannot in general determine necessary and sufficient conditions, conditions resembling these prototypical cases should qualify as disorders. In other words, not similarity connected to a set of necessary and sufficient properties, but global similarity to a prototype determines membership. While this strategy may at first sight avoid a whole range of problematic issues, it is in itself problematic, because, as Fulford [[36] p. 419] notes, it ducks major issues by opting out. Another issue is that Lilienfeld and Marino [20] do not distinguish between Roschian and vague concepts. Many of our concepts are vague, without firm boundaries. There is always the possibility that a boundary of a concept is vague because we are ignorant of underlying factors, which, if known, would provide a precise boundary. Additionally, it might very well be that the necessary and sufficient conditions are also vague [[37] p. 378]. Importantly, the vagueness of a concept does not warrant the conclusion that it is of Roschian structure and that necessary and sufficient conditions for category membership cannot be provided. Drawing on Wakefield [37] we could use the concept 'bachelor' to contrast vague and Roschian concepts. The concept 'bachelor' is vague: necessary and sufficient conditions for group membership can be given (unmarried, adult and male), but they are vague, since it is, for example, unclear at exactly what age a boy counts as a man. The same line of thought also applies to 'mental disorder'. Thus, the vague boundaries of a concept like mental disorder cannot by itself count as evidence against the possibility of there being necessary and sufficient conditions for category membership.

#### b) Psychopharmacological taxonomy

With the rise and rapid development of psychopharmacology, an alternative causal approach to psychiatric classifications emerged that aims at remapping psychiatric categories, depending on psychopharmacological effects. Such a view became known through the work of Peter Kramer on depression and Prozac [38]. Instead of merely treating patients, Kramer suggests that Prozac should be used as diagnostic tool. The underlying idea of such an approach is as follows: if fluoxetine successfully alleviates the symptoms of patients suffering from obsessive-compulsive disorder, anxiety disorders, depression, or premenstrual dysphoric disorders and distress, then the conclusion is warranted that each of these diseases have a common underlying cause. With psychopharmacological products as tools, Kramer suggests that we redraw the taxonomic map, providing the possibility of reconsidering connections between currently distinct mental disorders. In regard to the topic of this paper, one might object that psychopharmacological taxonomy is not pertinent. The objection could maintain that this taxonomic approach is only used for the categorisation of mental disorders and not such much in order to define 'mental disorder'. However, the idea that a mental disorder is a condition that psychopharmacological products have an alleviating effect on, underlies the *psychopharmacological taxonomy *approach. But just how consistent is this?

The first objection points to one the most severe problem of such an approach. Simply, the psychopharmacological approach cannot be of help in defining mental disorder: the fact that a psychopharmacological product has an alleviating effect on a mental condition does not justify the conclusion that the condition that the drug alleviates is a mental disorder. For instance, it is often the case that anti-depressive drugs alleviate particular forms of sub-threshold anxiety. Yet, this empirical conclusion does not directly translate into an ontological one. In other words, it does not say anything about whether the specific from of anxiety was a mental disorder or not. Second, another objection concerns the issue of reliability. Wallace [39] has noted that even most physiologically based diseases like infectious diseases, such a psychopharmacological approach would fail for at least two reasons: first, the same infection in different individuals does not always respond to the same medicine. Second, things are additionally complicated by the fact that distinctive infections may respond to the same medicine. To illustrate with the case of depression, the assumption that psychopharmacological taxonomy is that if an anti-depressant agent is effective on a depressed mood state then the depressed mood was caused by whatever chemical imbalance that the anti-depressant has proved effective on. However, as Radden [[40], p. 40] argues this might not always be the case. First, a depressed mood can sometimes be alleviated by social activity or by the consumption of alcohol. This means that in the framework of psychopharmacological taxonomy, we would have to conclude first that the depressed mood was caused by the lack of social activity or simply by the lack of alcohol. Second, we would have to say that whatever conditions are effectively alleviated by the consumption of alcohol must belong to the same category. On the basis of these problems, we must conclude that psychopharmacological taxonomy is to be rejected on grounds of unreliability and incoherence. Regarding to taxonomy, Zachar [[6] p. 172] suggests that we should be "skeptical of some biological psychiatrists' claims that every disorder that responds to anti-depressant medication must be a variation of the same disorder." We can make a related point here: if psychopharmacological taxonomy cannot provide a reliable re-definition of classification boundaries, it most likely will not provide aid in defining mental disorder.

## Conclusion

I have started out by saying that due to several socio-political factors, to many psychiatrists only a strictly objective definition of mental disorder, free of value components seems really acceptable. In this paper, I have explored and assessed a variant of such a naturalist approach to defining mental disorder, which I termed *natural function objectivism*. Such an approach aims to define a natural fact that underpins the definition of mental disorder. In order to objectively identify a particular condition as a disease, proponents of this approach make recourse to the notion of *natural function*. Central to this naturalist approach is the notion of natural function and the idea that value-dependent evaluations are not important to settle the issue whether something is dysfunctional. In general terms, proponents of natural function objectivism hold that while evaluation does trigger research interest, that after this preliminary evaluation the further study into the condition can be done objectively, without the involvement a value-laden judgments. As I see it, *natural function objectivism *comes in two varieties depending on the way the 'naturalness' of natural functions is understood. Guided by this distinction, the paper first explored the Christopher Boorse's 'biostatistical' account of natural function followed by an investigation of the merits of the '*hybrid naturalism' *approach to natural functions by Jerome Wakefield. I have concluded that the naturalist approaches can be divided into three categories depending on whether they attempt to define mental disorder merely as a natural kind, or as a biostatistical fact, an evolutionary dysfunction or adaptation have all revealed fundamental flaws. Due to the decisive difficulties of such 'natural function accounts' I argued that they fail to provide the backdrop for a reliable definition of mental disorder. Then, I have two proposals more or less explicitly call into question the whole effort of defining of metal disorder. While the first account argues that it is in principle impossible to provide necessary and sufficient conditions for membership in the category 'mental disorder,' the second account suggests that a definition of mental disorders should be based on psychopharmacology. At the end, both accounts showed crucial flaws in providing convincing reasons to reject the possibility of a good definition of mental disorder.

At the end, it seems that a convincing, wholly naturalist definition of mental disorder, involving no value judgments is impossible to find. But in what way does this pose an insurmountable problem? We have to remember that the pressure to find a naturalist definition emerged in a specific historical situation when psychiatry was under attack. It was under these particular circumstances that Boorse's and Wakefield's naturalist accounts attempted to define the domain of natural dysfunction as the proper domain of psychiatry [[41] p. 124]. Importantly, the basic assumption of such 'natural function objectivism' - that was used to attempt to resolve the fierce debates pertaining to the scientific status of psychiatry - is that a clear separation of natural (evolved and innate) and socially cultivated psychological functioning is possible. However, it might be the case that this underlying assumption of natural function objectivism is doubtful. In general, since then, both the socio-political situation and our scientific knowledge have substantially changed and the changed situation now calls for a revision of the basic dichotomy presupposed by both sides of the anti-psychiatry debate as well as by the naturalist solutions to the debate. As the socio-political background changes, it is evident that psychiatry no longer faces the same kind of harsh criticism of being a political organ. This is not to say that psychiatry no longer faces political critique. The point is merely that the criticism today is less uncompromising. As to the scientific changes, new developments at the nexus of genetics and psychology, the growing emphasis on gene-environment interactions indicate that psychological phenotypes are neither merely evolved functions nor are they created by environmental circumstances (including social ones) [[41] pp. 272-276]. Rather, they are products of complex patterns of interaction between them. If this is the case, and current science is undermining the clear division between the natural (evolved) and socio-cultural, then such basic dichotomy presupposed by both sides of the anti-psychiatry debate as well as by the naturalist solutions to the debate must be revised. As it seems, the kind of medical norm that is relevant in psychiatry cannot be defined as a domain on its own, apart from the social. As Kirmeyer [[42] p. 19] rightly points out, even our very idea of 'natural' adaptation is culturally biased: "we tend to think of adaptation in terms of individuals rather than groups because of the individualistic bias of Euro-American societies, and we are less likely to recognise and give central place to functions whose main purpose may be intragroup harmony rather than individual fitness."

All in all, we need more attention to the fact that besides being evolved, psychological functions are also realised in specific cultural contexts and in specific individuals. Instead of attempting to abstract from these contexts of realisation, a concept of mental disorder must acknowledge and reflect the multiple and interweaving origins of design (the genetic, the social and the individual).

In the beginning of this paper I noted that within the framework of the development the DSM-V, researchers have once more directed focus on the concept of mental disorder. There is considerable disagreement on this issue. While some [8] argue not only to retain but also to additionally emphasise the "dysfunction" criteria in the DSM-V, others [1] seek for credible alternatives for this term. Due to the problematic issues with naturalistic concept of dysfunction that this paper has elaborated, I suggest that efforts should be made towards finding alternatives that are not directly associated with particular theories of natural function. Both for this reason, and in order to attempt to maintain the theory neutrality of the DSM-V, the best alternative might be a different term that is not directly associated with any particular theory of function.

## Competing interests

The author declares that they have no competing interests.

## About the Author

Somogy Varga is a postdoctoral researcher in Philosophy of Mind and Cognition at the Institute of Cognitive Science at the University of Osnabrück and currently a visiting researcher at the Center for Subjectivity Research in Copenhagen. Previously, he has worked at the Institute of Social Research in Frankfurt and received his Ph.D. from the University of Frankfurt with a dissertation on social philosophy (supervised by Axel Honneth). He mainly pursues research interests in the philosophy of psychiatry, philosophy of mind, phenomenology, social philosophy and critical theory.
